# Epidemiological analysis of occupational dermatitis notified in Brazil in the period 2007 to 2012*

**DOI:** 10.1590/abd1806-4841.20164762

**Published:** 2016

**Authors:** Gabriela Yumi Plombom, Mariana Santos de Oliveira, Fernanda Lika Tabushi, Amanda Joekel Kassem, Kátia Sheylla Malta Purim, Renato Mitsunori Nisihara

**Affiliations:** 1 Universidade Positivo – Curitiba (PR), Brazil

**Keywords:** Dermatitis, occupational, Occupational exposure, Occupational health

## Abstract

**BACKGROUND:**

Occupational dermatitis affects the quality of life and productivity of workers. Studies on the subject are scarce in Brazil. It is estimated that the disease is underreported and that many affected patients do not seek health care.

**OBJECTIVES:**

To conduct an epidemiological analysis of occupational dermatitis notified via SINAN in Brazil from January 2007 to December 2012; evaluate the profile of patients assisted; and check the main etiological agents involved.

**METHODS:**

We analyzed the compulsory notification forms of cases of occupational dermatitis filled nationwide during January 2007 to December 2012.

**RESULTS:**

During the study period 3027 cases of occupational dermatitis were notified in Brazil. In 61.4% of cases patients were men aged between 35-49 years (39.6%). The most described etiological agent was chromium (13.9%). The location of the body most affected was the hands, with 28.4% of cases. The construction sector is implicated in 28.7% of cases and domestic services by 18%. Allergic contact dermatitis is the most prevalent occupational dermatitis (20.6%) and the region with the highest number of notifications was the Midwest, with 376.4 cases per million inhabitants.

**CONCLUSIONS:**

The profile of patients most affected by occupational dermatitis in Brazil during the study period was: men with elementary school, aged between 20 and 49 years old and working in the construction industry. The most common occupational dermatitis were allergic contact dermatitis caused by chromium after years of exposure, being the hands and head the parts of the body most affected.

## INTRODUCTION

Conceptually, occupational dermatitis (OD) are defined as skin, mucous and attachments changes directly or indirectly caused, conditioned, maintained or aggravated in professional activity or work environment. The etiologic agents are varied and classified into biological, physical or chemical.^1,2^ Studies on the subject are scarce in Brazil, and the real risk factors and the prevalence of the disease in the country are unknown. However, it is known that the disease is underreported and that many affected workers do not seek treatment.^3^ The quality of life and productivity of workers can be affected by skin diseases, increasing the allocation of resources, either by the employer, employee and/or the health system as a whole.

From 2002, within the National Network of Integral Attention to Worker Health (RENAST), it was established that all ODs were notified by completing compulsory notification forms, orientation regulated by Ordinance 777, in April 28th 2004, to all cases treated in a hospital setting or in health facilities.^4^ This measure aims to increase knowledge about the main harm to workers' health.

Considering the need for the availability of consistent information on the profile of workers and the occurrence of work-related ODs to guide health actions and intervention in the working environment and conditions, the objective of this study was to conduct an epidemiological study of the ODs notified via Notifiable Diseases Information System (SINAN) in Brazil, from January 2007 to December 2012, evaluate patients' profile, and identify the main etiological agents involved in these cases.

## METHODS

This study was approved by the Institutional Review Board under the number 667599/2014. This is a descriptive cross-sectional study in which data from compulsory notification form (CNF) contained in SINAN, in the period between January 2007 and December 2012, were collected retrospectively. The data were accessed through the Department of Labour Medicine of the Hospital do Trabalhador de Curitiba-PR. The sample of this study is characterized as non-probabilistic, with intentional character and sequential selection.

The CNFs have been implemented and must be manually filled in hospitals or local health units by previously trained professionals. Subsequently, they are scanned and sent to SINAN's national database.

In total, 3,027 records of ODs cases were analyzed throughout the national territory during the period investigated. The notification forms comprise the following data: age, gender, education, federal unit and occupation of the patient; main dermatitis causative agent, lesion site, completion of the epicutaneous test, ICD-10, time of absence from work, evolution of dermatitis and issuing of Work Accident Communication (CAT). ICD-10 codes were separated according to the classification of Alchorne *et al.* (2010); occupations were divided into economic sectors; and records by state were grouped into regions for better comparison. The division of the number of cases per million of inhabitant was calculated using the 2010 demographic data of IBGE website.^5^ All the records that did not match the specified period were excluded from the analysis.

Data were organized and tabulated in spreadsheets using Excel (Microsoft Office, version 2013), applying descriptive statistics with the help of statistical program Graph Pad Prism, version 5.0.

## RESULTS

**Graph 1:**
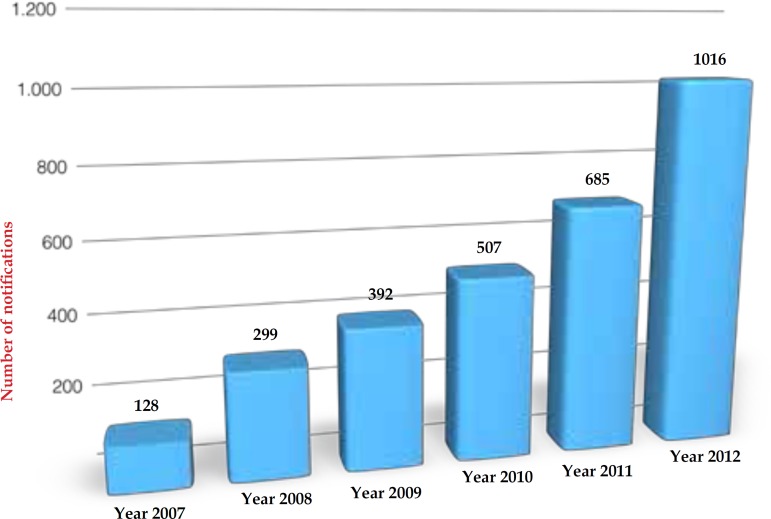
Number of completed compulsory notifications from January 2007 to December 2012 (n = 3,027)

From January 2007 to December 2012, a total of 3,027 patients were diagnosed with ODs. Graph 1 shows the increase in the number of notifications over the years.

In terms of age, 72.4% of patients were aged between 20 and 49 years. Men were predominant in notifications, with 61.4% (1,859/3,027) of cases, while women represented 38.5% of the total (1,167/3,027), and only one case was identified as ignored. Regarding education, primary education was predominant (44.9%) and only 4.1% had higher education. Demographic data are shown in table 1.

**Table 1:** Demographic data of the patients studied

	N=3,027	%
**Age**		
0-14	33	1
15-19	91	3
20-34	993	32.8
35-49	1.201	39.6
50-64	590	19.4
65-79	95	3.1
80>	24	0.7
**Education**		
Ignored / Blank	586	19.3
Illiterate	73	2.4
Elementary school	1.361	44.9
High school	878	28.9
Higher education	129	4.1
**Economic sector**		
Construction sector	871	28.7
Domestic service	546	18
Environmental and agricultural sciences	411	13.5
Management and business	273	9.0
Health	252	8.3
Industry	227	7.4
Other	219	6.9
Hotel/Restaurant	115	3.7
Ignored	113	3.6

About the division into economic sectors, it was noted a predominance of the construction sector with 871 (28.7%) cases of ODs, followed by 546 (18%) notifications in the domestic service area (Table 1).

Table 2 presents data on the location and type of dermatitis agent described in CNFs. We found that the hand was the place most affected by ODs, with 28.4% of cases, followed by the head, with 20.1%. Regarding the causative agent of lesions, 421 (13.9%) were caused by chromium, 143 (4.7%) by woods and 107 (3.5%) by solvents. It is noteworthy that in 2,057 (67.9%) cases, the field for the causative agent of the lesion was blank or was filled as "ignored". Regarding the reported diagnosis, allergic contact dermatitis (ACD) was the most frequent among the ODs, totaling 20.6% of cases. Secondly, there are keratoses, 17.2%, followed by ODs, with 16.5% of patients. In other 15.3% were included all ICD-10 that did not correspond to skin changes (poisoning, trauma, asthma, cysts, conjunctivitis, etc.) or that did not fit the previously stipulated classification (burns, psoriasis, radiodermatitis, erythema multiforme, melasma, etc.). Irritant contact dermatitis (ICD) totaled 11.5% of diagnosis and unspecified dermatitis, 5.6% of notifications.

**Table 2:** Data on reported dermatitis

	N=3,027	%
**Lesion site**		
Hand	862	28.4
Head	610	20.1
Other	376	12.2
Ignored/Blank	316	10.4
Upper limb	306	10.1
Whole body	291	9.6
Lower member	266	8.7
**Agent**		
Ignored/Blank	2.057	67.9
Chromium	421	13.9
Other	299	9.5
Wood	143	4.7
Solvents	107	3.5
**Dermatitis**		
Allergic Contact Dermatitis	626	20.6
Keratosis	522	17.2
Occupational dermatoses	502	16.5
Other	470	15.4
Irritant Contact Dermatitis	350	11.5
Unspecified dermatitis	172	5.6
Infections	146	4.8
Ignored/Blank	143	4.7
Onichia	88	2.9
Acnes	8	0.2

In table 3, data on exposure time and evolution of reported ODs are displayed. In 51% of cases, the exposure time was reported as years; in 21.8%, the field was completed as ignored or left blank; and in 12.7%, the time was reported in months. The absence from work due to dermatitis was necessary for 975 patients (32.2%); 1,441 (47.6%) workers did not need work leave; and the option ignored/blank was found in 611 records (20.1%). During the study period, it was identified only one death; 628 cases (20.7%) developed with healing; 66 (2.1%) developed with partial permanent disability; and 7 (0.2%) with total permanent disability.

**Table 3:** Clinical data of dermatitis

	N=3,027	%
**Exposure time**		
Ignored/Blank	662	21.9
Hours	247	8.1
Days	189	6.2
Months	385	12.7
Years	1.544	51
**Evolution**		
Other	949	31.3
Cure	628	20.7
Ignored/Blank	616	20.3
Temporary disability	521	17.2
Unconfirmed healing	239	7.8
Partial permanent disability	66	2.1
Total permanent disability	7	0.2
Death by occupational disease	1	0.03

Although recommended, the epicutaneous test was performed in only 19.8% (598) of the patients and it was not performed in 29.4% (889). In 50.8% (1,540) of notifications, this information has been marked as ignored or left blank. The issue of CAT was performed in 19.4% (590) of cases.

Table 4 shows the distribution by region in Brazil, verifying that the greatest frequency of cases occurred in the Midwest region, which notified 376.4 cases per million inhabitants. In the South there was the lowest number of ODs notifications, with 18.7% of cases.

**Table 4:** Demographic distribution of dermatitis

Regions	N cases per million inhabitants
Midwest	376.4
Southeast	123.4
Northeast	38.8
North	26.9
South	18.7

## DISCUSSION

Epidemiological data on occupational causes of diseases are always relevant, since they reflect the quality of assistance to workers' health, improve the knowledge on the major diseases that are subject to such people and enable that preventive measures are implemented.

During the study period (six years), there was significant increase in the number of notifications over the years, which may be explained by population growth, increased demand for labor, greater exposure to causative agents of disease, creation of workers' health protocols and greater access to primary care networks.

In the present investigation, ODs were more frequent in men, aged between 20 and 49 years, with elementary education and working in the construction sector. These findings are similar to those observed by other authors in Brazil and in other countries.^3,6-9^ It is suggested that these findings are due to the fact that young workers are less experienced, more impulsive and used to acting with less caution in handling potentially hazardous chemical agents to the skin.^3^ The construction industry employs many people and presented increased activity in recent years. From 2003 to 2009, the mean growth rate of formal construction companies in Brazil was 11.2% per year, which is more than twice the industry rate (5.1% per year). Eventually, young people were hired, without a lot of experience and with little trained for the tasks. ^10^

According to the European Risk Observation Report, the hand was body region most affected by the ODs, with 80% of cases.^8^ In our study, this finding was also observed, with the hand occupying the first place, with 28.4% among the affected sites. This result suggests that there may occur a lack of use of PPE (Personal Protective Equipment) by workers, such as gloves, or we can also conclude that the quality of PPE is not satisfactory. In addition, in this sector, there is frequent contact with causative agents such as chromium, rubber and wood.^3^ The domestic service sector was second in occurrence of dermatitis, probably due to excessive contact with moistures, soaps and detergents, cleaning products and use of rubber gloves. ^11^

Regarding dermatitis, in this study it was observed 1,148 cases of dermatitis were reported, and the ACDs were the most common (20.6%). Goon *et al.*, in Singapore, described the ICDs as the most incidents (61.2%), and the ACDs, with an incidence of 36%.^6^ In Europe, it is also noted that ICDs (80%) prevail over DCAs (10%).^8^ It is noteworthy that, for the etiological agents in this investigation, chromium was the main agent identified. The presence of hexavalent chromium in the wet cement (the most widely used in civil construction in Brazil) is an abrasive and alkaline agent that may predispose to ACD.^3,7^ The use of rubber gloves on wet or previously damaged skin can also promote the development of ACD.^3^ Lazzarini *et al.* (2012) conducted a study with 525 subjects, of which 53 were masons, confirming through the epicutaneous test the prevalence of ACD in 76% and of ICD in 24% of this population.^7^ Other authors, in a study conducted in Europe, found as main agents detergents allergens, solvents and oils, and the most affected workers were in sectors of the metal industry, followed by workers in construction and transport sectors. The economic sector with the highest number of cases is the manufactured sector (10.4%), followed by the construction sector (9.1%).^8^ The completion of the epicutaneous test is essential in the investigation of ACD and in the identification of the causative agent.^3,12^ However, the test is time-consuming - the reading is done after 96 hours - and needs technical training, material, method, time, interpretation and proper care in its application. In Brazil, its use is still limited and requires improvements.^13^ In our study, of the total cases of dermatitis (n=1,148), the epicutaneous test was performed in only 19.8% (598) of reported cases.

Generally, chemical agents are responsible for 80% to 90% of dermatological diseases.^8^ In this study, in 67.9% of the notifications, the field for the main causative agent of dermatitis was left blank or was filled as "ignored". In Germany, Diepgen (2012) estimated that 23,596 workers were affected by ODs in 2010 and about 80% were involved in the following groups: hairdressers, metalworkers, health professionals, cooks, builders, cleaning crews and painters. The main aggravating agent found was the water, since it propitiated wet conditions that predisposed the irritative dermatitis. Also, the frequent use of waterproof gloves can cause abrasions and does not allow evaporation of sweat.^9^

In Greece, Zorba *et al.* (2013), studying 4,000 workers, identified 39.9% cases of ODs in this population, reporting that the highest rates of prevalence of skin lesions were found in workers of the metal industry, wood industry, automotive industry, construction industry and also cooks (in the automotive and construction industries, all affected individuals were men).^14^

Regarding the time of exposure to the causing agent, most cases (51%) reported in Brazil was reported as years of contact, which may also explain the prevalence of ACD in relation to the ICD because the ACD require at least a week to raise awareness and occur after months or years of contact.^3^ In the evolution of ODs, it was reported cure in 20.7% of assisted patients, temporary disability in 17.2% of cases and permanent disability in 2% of cases (Table 3). The economic impact of the disease is inevitable, treatment may be long and eventually the withdrawal of the occupation is necessary. In some cases it may also be necessary the process of rehabilitation and re-adaptation to work. The information obtained, combined with protective and preventive measures, will contribute to improving the quality of life and productivity of workers.

The region with the highest number of reported cases was the Midwest, with 376.4 cases per million inhabitants. Possibly this was the region where there was increased surveillance and control of the need for notification, but not necessarily higher incidence of ODs. It is still necessary that all states of the federation are more charged and committed to adopting the notification form. Moreover, it is important to qualify the professionals who treat affected patients and guide companies so their employees seek health services when they have some skin disease that may be related to the work environment.

The study has limitations mainly related to factors such as failure to notify ODs in Brazil. It is assumed that many cases of skin diseases related to the work environment are treated without the demand for health services or professionals working in the company, not occurring notification. On the other hand, when the ODs are notified, it was noted that inadequate filling in the forms recurs in all units of the federation. Some fields containing information that would be of utmost importance, especially regarding the causative agent of ODs, were filled as "ignored" or left blank in 67.9% of cases.

The SINAN app is designed to store, from tools and standardized access codes at national level, the information of notifiable diseases, through their respective notification forms. However, for SINAN fulfill its objectives, the awareness of health workers to properly fill the CNFs is needed.

## CONCLUSION

The profile of patients most affected by ODs in Brazil in the period studied was: men with elementary school, aged between 20 and 49 years old, and working in the construction industry. The most frequent ODs were allergic contact dermatitis caused by chromium after years of exposure, and the hands and the head were the parts of the body most affected.

There was also the need for more information and demand for the proper filling of the CNFs.
